# The Sound of Emotion: Pinpointing Emotional Voice Processing Via Frequency Tagging EEG

**DOI:** 10.3390/brainsci13020162

**Published:** 2023-01-18

**Authors:** Silke Vos, Olivier Collignon, Bart Boets

**Affiliations:** 1 Center for Developmental Psychiatry, Department of Neurosciences, KU Leuven, 3000 Leuven, Belgium; 2 Leuven Autism Research (LAuRes), KU Leuven, 3000 Leuven, Belgium; 3 Leuven Brain Institute (LBI), KU Leuven, 3000 Leuven, Belgium; 4 Institute of Research in Psychology & Institute of Neuroscience, Université Catholique de Louvain, 1348 Louvain-La-Neuve, Belgium; 5 School of Health Sciences, HES-SO Valais-Wallis, The Sense Innovation and Research Center, 1007 Lausanne and 1950 Sion, Switzerland

**Keywords:** emotion discrimination, voice, frequency-tagging, electroencephalogram

## Abstract

Successfully engaging in social communication requires efficient processing of subtle socio-communicative cues. Voices convey a wealth of social information, such as gender, identity, and the emotional state of the speaker. We tested whether our brain can systematically and automatically differentiate and track a periodic stream of emotional utterances among a series of neutral vocal utterances. We recorded frequency-tagged EEG responses of 20 neurotypical male adults while presenting streams of neutral utterances at a 4 Hz base rate, interleaved with emotional utterances every third stimulus, hence at a 1.333 Hz oddball frequency. Four emotions (happy, sad, angry, and fear) were presented as different conditions in different streams. To control the impact of low-level acoustic cues, we maximized variability among the stimuli and included a control condition with scrambled utterances. This scrambling preserves low-level acoustic characteristics but ensures that the emotional character is no longer recognizable. Results revealed significant oddball EEG responses for all conditions, indicating that every emotion category can be discriminated from the neutral stimuli, and every emotional oddball response was significantly higher than the response for the scrambled utterances. These findings demonstrate that emotion discrimination is fast, automatic, and is not merely driven by low-level perceptual features. Eventually, here, we present a new database for vocal emotion research with short emotional utterances (EVID) together with an innovative frequency-tagging EEG paradigm for implicit vocal emotion discrimination.

## 1. Introduction

We hear sounds every day, everywhere [1]. Being able to discriminate these sounds, contributes to a better understanding of the world around us. The human voice is by far the most socially relevant and familiar sound category for human beings [2]. Besides the specific linguistic content, the human voice offers a lot of socio-communicative information about the speaker. For instance, in a wink, it gives us an idea about the gender, approximate age, and the emotional state of the speaker [3,4,5]. Additionally, when listening carefully, one may even extract more subtle speaker information, such as the speaker’s personality (e.g., extravert versus introvert) or the speaker’s demographic origin [6,7]. Efficient processing of all this supra-linguistic information is required to successfully engage in social communication.

### 1.1. Vocal Emotion Processing as a Gateway to Social Communication

While zooming in on vocal emotional processing, speech prosody provides important cues about the emotional state of our conversational partners. Similar to the visual face processing domain [8], it has been postulated that a restricted group of so-called “basic” emotions (happy, surprise, angry, fear, sad, and disgust) can be universally recognized across different cultures when vocally expressed, even without the presence of linguistic meaning [9]. Supporting this idea of basic emotions, a meta-analysis on the neural correlates of vocal emotion processing revealed that these basic emotions are distinct and characterized by particular patterns of brain activity [10].

The recognition of vocally expressed emotions happens automatically [11]: we cannot inhibit recognizing an emotion in a voice, for instance when talking to someone who recently got fired or, in contrast, who just got a promotion, we can identify the emotional state of this person as sad or happy within a few hundred milliseconds, even without any linguistic context. Emotion recognition also happens extremely fast and based on limited auditory information. An ERP study demonstrated a neural signature of implicit emotion decoding within 200 ms after the onset of an emotional sentence [12], suggesting that emotional voices can be differentiated from neutral voices within a 200 ms timeframe. Explicit behavioral emotion recognition may take a bit longer, ranging from 266 to 1490 ms, depending on the paradigm and the particular emotion [13,14,15]. The fast decoding of emotion prosody is not only found in humans but is also visible in a variety of other animals, which indicates that recognizing emotions from voices is an important evolutionary skill to communicate with conspecific animals [16].

Gating paradigms have indicated that different vocal emotions are recognized within a different time frame (e.g., fear recognition happens faster than happiness), thereby suggesting that the fast recognition relies on emotion specific low-level auditory features [14]. Vocal emotion categories are indeed characterized by particular auditory features. For instance, sad speech is generally lower in pitch, and this is the case across different languages and cultures [17]. A classical, but almost intrinsically paradoxical challenge in vocal emotional neuroscience, is the demonstration that emotion discrimination is not purely driven by low-level acoustic cues [18,19,20]. This echoes the broader attempts of demonstrating that (emotional) voice processing and the selective neural activity in the so-called temporal voice areas is not merely determined by particular spectro-temporal acoustic characteristics, often accomplished by a rigorous matching of vocal versus non-vocal low-level cues [21]. Besides determining the basic low-level acoustic cues that characterize and classify vocal emotions, there is evidence that threat related vocal signals mostly attract our attention, even when basic voice acoustics are comparable with non-threat related emotional vocalizations [22]. This indicates that low-level cues alone do not fully capture the experience of the vocally expressed emotions.

The temporal voice areas are located in the middle part of the auditory brain; these areas respond preferentially to voices compared to non-vocal environmental sounds [23]. This selective sensitivity for voices is particularly pronounced in the right hemisphere. Moreover, these temporal voice areas respond stronger to utterances spoken in an emotional rather than neutral tone [3,21,24,25]. The classical rightward lateralization of emotional voice processing was challenged by Kotz et al. (2003) [26] who demonstrated that increasing task demands also resulted in an increasing left lateralization. In terms of lateralization of processing low-level acoustic features, pitch and slowly fluctuating signals have been shown to be processed preferentially at the right side whereas shorter and faster temporal information is typically processed in the left auditory cortex [27,28,29]. Given the critical importance of pitch to differentiate vocal emotion categories, this might explain that the majority of studies observe a right side lateralization for emotional voice processing [30].

As outlined above, it is evident that efficient emotion processing—including vocal emotion processing—is crucial for social functioning. Many psychiatric disorders are characterized by difficulties in social functioning, including emotion processing abilities, with key examples in autism spectrum disorder, schizophrenia, and anxiety and mood disorders (for reviews, see [31,32,33,34]). Thus, assessing individual differences and deficits in sensitivity for socio-communicative emotional cues is central in clinical practice, but objective and reliable diagnostic instruments are lacking, especially those tapping automatic emotional processing. A series of semi-standardized behavioral socio-cognitive tasks have been developed, assessing emotion recognition abilities for vocal, facial, and bodily expressive stimuli (e.g., [35,36,37]). Yet, generally, these tasks do not differentiate sensitively between clinical and neurotypical populations, often because they allow the mobilization of alternative compensatory perceptual and cognitive strategies [38,39].

Brain imaging studies on the other hand show more robust group differences in vocal emotion processing. The auditory mismatch negativity (MMN), for example, is an event related potential component that reflects the response to an auditory deviant sound. This component is frequently used to investigate group differences in emotion processing. For instance, Schirmer et al. (2005) [40] demonstrated reduced MMN responses to emotionally deviant sounds in men as compared to women, and Lindström et al. (2018) [41] suggested that MMN components could indicate impaired emotional prosody perception in individuals with autism spectrum disorder. However, MMN studies lack high signal-to-noise ratio, thereby necessitating long recording sessions and reducing the utility to characterize performance at the individual subject level [42]. This has clear consequences for research with clinical populations or even infants.

Accordingly, there is a need for instruments that allow objective and robust assessment with high signal to noise responses of automatic and implicit emotion processing abilities, reliable at the individual subject-level, and preferably within a short timeframe. Here, we propose that frequency-tagging EEG in combination with periodic auditory (vocal) stimulation offers this approach, and we present evidence that the brain selectively responds to emotional vocal cues embedded within a stream of neutral vocal utterances.

### 1.2. Frequency Tagging EEG to Pinpoint Differences in Socio-Communicative Abilities

Recently, it was demonstrated that fast periodic visual stimulation combined with EEG can be used as an implicit neural index of the sensitivity for subtle socio-communicative facial cues, such as facial identity and facial expression [43,44]. Application of this innovative approach in clinical populations (e.g., autism spectrum disorder and velocardiofacial syndrome), allowed pinpointing subtle but robust deficits in socio-communicative sensitivity that otherwise remained concealed via classical behavioral face processing tasks [45,46,47,48]. A more recent pioneering study applied this same frequency-tagging EEG approach with auditory stimulation, thereby demonstrating that voices can automatically be differentiated from both non-vocal environmental sounds and music instruments with highly similar low-level features [49]. Proceeding from this seminal study, here, we will extend this frequency-tagging EEG approach and apply it for the first time to investigate vocal emotion processing. In particular, we will characterrize the neural signature of automatically detecting periodic emotional vocal utterances among a stream of neutral vocal utterances, and we will explore to what extent this neural discrimination ability is driven by the socio-emotional characteristics of the stimuli or by more basic low-level acoustic differences between the stimulus categories.

To investigate if our brain can systematically track a stream of emotional vocal utterances within a standard stream of neutral vocal utterances, we designed a Fast Periodic Auditory Stimulation (FPAS) paradigm and combined it with scalp EEG recordings. The basic principle of this frequency-tagging approach is that the periodicity of the electrophysiological response on the human scalp corresponds exactly with the periodicity (frequency) of the auditory stimulation. We used an oddball paradigm where standard sounds were presented at a base rate frequency of 4 Hz and oddball sounds were inserted periodically into the sequence every third sound. In particular, neutral voices were presented at a 4 Hz base rate and emotional voices (angry, sad, happy, and fearful, in separate paradigms) were presented at a 1.333 Hz oddball rate. Whenever a change (i.e., discrimination between the neutral and the emotional utterances) is perceived, in addition to the periodic response to the base rate, a periodic response corresponding to the presentation frequency of the emotional voices (i.e., 4/3 = 1.333 Hz) is also observed. The main advantages of using this FPAS approach are: (a) the response can be measured implicitly, i.e., without an explicit behavioral task; (b) the response can be identified objectively since it occurs at a predefined frequency; (c) it can be quantified directly by comparing the response at that frequency (signal) with responses at neighboring frequencies (noise); and (d) the technique is extremely robust, since it is immune to artefacts and yields high signal-to noise ratio (SNR) responses in a short amount of time, which makes it suitable for clinical populations (for a review, see [50]).

## 2. Materials and Methods

### 2.1. Participants

We recruited 20 male participants for this study (mean age = 25.19 years, SD = 4.08, range = 19–34, all right-handed); the sample size was based on previous fast periodic auditory stimulation studies (e.g., [49]). We only included male participants to avoid gender effects in the recognition of vocally expressed emotions [51]. All subjects reported intact hearing ability, which was confirmed by pure tone audiometry (average PTA hearing loss below 25 dB HL for every participant). Subjects were Dutch native speakers and received a monetary reward for participating. No one reported any history of psychiatric or neurological disorders. Before the start of the experiment, all subjects signed an informed consent form approved by the Medical Ethics Committee UZ/KU Leuven (reference S62969).

### 2.2. Stimuli: Design of the Emotional Voices and Identity Database (EVID)

**Figure 1:**
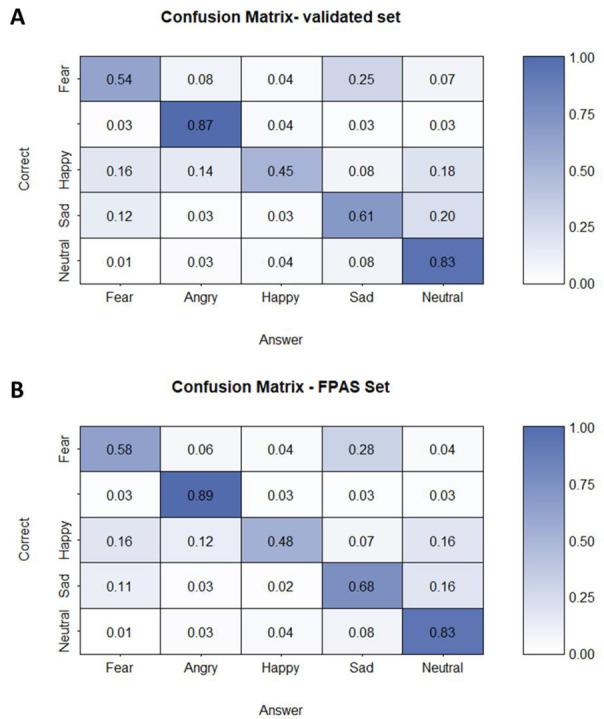
(**A**) Confusion matrix of the 500 emotional vocal stimuli. The rows indicate the presented emotional stimulus category (correct), the columns indicate the provided response (answer). The numbers indicate the proportional responses, averaged across the 40 participants. The diagonal shows the proportion of correct answers for each emotion. (**B**) Matrix of the stimuli used for the FPAS paradigms.

For the FPAS trials we created a new, large, and well-controlled voice segments database, incorporating all the stimulus features that are relevant for our research objectives. We aimed for short clips with a recognizable emotional value, while also demonstrating large variability across other sound features such as pitch, harmonic ratio, phonetic content, and speech rate. All voice segments were extracted from the Crowd Sourced Emotional Multimodal Actors Dataset [52], which encompasses audio and video recordings of 13 short sentences, spoken by 48 male and 43 female actors, according to six emotional states (neutral, happy, angry, sad, fear, and disgust). We extracted 3960 short 250 ms utterances from these emotionally pronounced sentences (20 actors × 33 utterances × 6 emotions). Utterances were cut at the beginning of a randomly chosen phoneme. Thus, depending on speech rate, word length, and phoneme position, these 250 ms utterances resulted in words (e.g., get) and non-words (e.g., ge). Each utterance started and ended with a linear fade in and fade out of 10 ms to avoid clipping of the sounds. All utterances were equalized in overall energy (RMS). We validated the stimuli behaviourally in a separate sample of 40 healthy young adults (age = 18–35 years old) to examine which stimuli are categorized best in terms of emotion, and we maintained a subset of 500 stimuli that are categorized most consistently. The subset contains a set of 10 speakers (five female and five male speakers), each pronouncing 10 different phonetic utterances of 250 ms with a 10 ms fade in/out according to five emotion categories (neutral, happy, angry, sad, and fear). Note that these utterances were not the same over all emotions as we selected the utterances with the highest recognition rate. See Figure 1 for the confusion matrix. We refer to this newly designed and validated emotional stimulus set as the EVID (Emotional Voices and Identity Database), which is available upon request by the first or senior author.

### 2.3. Procedure and Equipment

**Figure 2:**
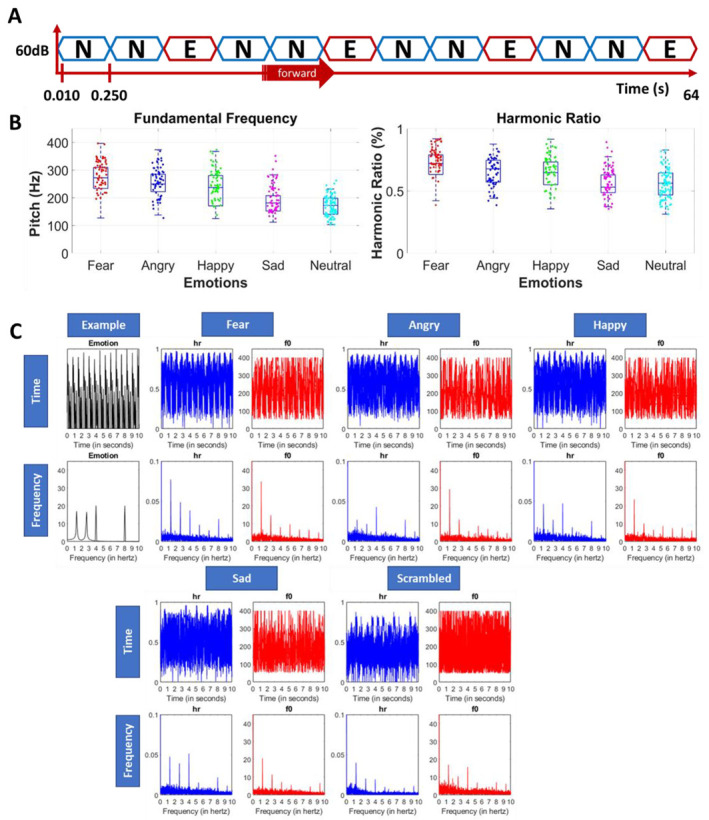
(**A**) Schematic representation of the paradigm. The figure shows a base rate frequency of 4 Hz, with emotional stimuli (E) being interleaved every third stimulus, hence at 1.333 Hz. (**B**) Low-level features of the vocal utterances. Low-level features are plotted for every single stimulus of every emotion condition. On the left the pitch (f0, fundamental frequency) is plotted and on the right harmonic ratio (hr in %). Note the large variability within every stimulus category (see Supplementary Figure S1 for the scrambled stimuli). (**C**) Periodicity. The periodicity of the low-level features across the entire acoustic sequence. The first box represents a symbolic preview of the first 10 s of a sequence and shows the presence of the emotional oddball stimuli in the time domain (s) and in the frequency domain (Hz), with the clear 4 Hz peak indexing base rate and the 1.333 Hz peak indexing the emotional oddball stimuli (in black). Next, we plotted for all sequences of every emotion category as well as for the scrambled control condition the variability in harmonic ratio and pitch in the time domain and the frequency domain. This analysis reveals that, despite inducing a huge amount of acoustic variability, for all conditions (including the scrambled one), the low-level features are periodically preserved in the frequency domain, both at the base and at the oddball rate.

For each of the emotions (i.e., happy, angry, sad, and fear), we created an oddball paradigm where the emotional utterance was periodically presented in a stream of neutral utterances. The 250 ms duration of the utterances naturally leads to the 4 Hz base frequency of the sound presentation, and the emotional utterances were interleaved every third stimulus, leading to an oddball frequency of 1.333 Hz (i.e., NNENNENNE…, see Figure 2A). For every condition (i.e., emotion), we created six sequences, each uttered by a different speaker (including three sequences with a female speaker and three with a male speaker). For each condition we used the speakers with best recognition rate for the emotion in question (see Figure 1 for the confusion matrix of the used utterances in this study). Note that in every sequence the same speaker was used for the neutral and for the emotional utterances. The sequences were 64 s and had a linear fade in/out of 2 s.

In addition to the four emotion category conditions (happy, angry, sad, and fear), we created a scrambled control condition with similar low-level acoustic characteristics but without the emotional content. We scrambled the sounds of the four emotion categories and the neutral emotion category based on the method of Dormal et al. (2018) [53], which results in sounds with equal frequency content and spectral-temporal structure as the original sounds, but with a different harmonicity. This ensures that the emotion category is no longer recognizable in the scrambled sounds, while the low-level acoustic cues are largely preserved. We created six scrambled control sequences with three male and three female speakers, covering the four emotion categories.

Figure 2B provides an overview of the acoustic characteristics of the vocal stimuli included in the experiment, illustrating that all stimulus categories are highly heterogenous in terms of pitch and harmonic ratio, and that the differences within an emotion category are much larger than the average differences between the emotion categories. Pitch is defined as the fundamental frequency (f0) of the utterance and refers to the perception of the sound as relatively high or low (see Supplementary Figure S1 for the scrambled stimuli). Here, it has been calculated by means of the MATLAB function pitch (audioIn, fs). Harmonic ratio involves the ratio of the fundamental frequency’s power to the total power in an audio fragment and refers to the degree of harmonicity contained in a signal. Here, it has been calculated by means of the MATLAB function harmonicRatio (audioIn, fs). Yet, as expected and despite our attempts to induce as much natural variability as possible, neutral, and emotional utterances are not perfectly matched for low-level acoustic features. To further investigate the impact of these low-level features on the periodic neural oddball responses, we applied the following procedure: (a) we entered the wav-file of the entire acoustic 6 × 64 s sequences in MATLAB and calculated the harmonic ratio using 100 ms rectangular windows with 50 ms overlap and pitch (Normalized Correlation Function for estimation of pitch) with a window length of 52 ms with 42 ms overlap, and (b) we transformed the continuous temporal signal from the temporal to the frequency domain by Fourier transformation to investigate the periodicity of these acoustic features (cf. [54]). As displayed in Figure 2C, one can see that despite the massive variation of the heterogenous stimuli, characteristic low-level features were still somehow periodically preserved in the stimulation sequences. Importantly, this low-level acoustic periodicity was not only preserved in all the vocal emotional sequences, but also in the control sequences with the scrambled stimuli.

We used an ActiveTwo Biosemi system with 64-Ag/AgCl electrodes and two additional electrodes as reference and ground electrodes (Common Mode Sense active electrode and Driven Right Leg passive electrode). Sound sequences were created and presented in a random order via a custom-built MATLAB script. Sounds were presented via a calibrated RME Fireface UC with Etymotic Research ER-1 insert earphones to make sure all sounds were presented at an equal intensity of 60 dB SPL. Participants listened to the sound sequences with eyes closed.

To ensure that participants stayed focused on the sound sequences, we included an orthogonal behavioural task, which was non-periodic and unrelated to the emotional value of the stimuli. This task involved detecting short 500 ms silence periods in the sounds stream, occurring randomly four times in every sequence (not in the first and last 5 s, and at least 5 s apart from each other). Participants had to press a button whenever they detected this silence.

### 2.4. EEG Analysis

#### 2.4.1. Pre-Processing

We used the Letswave6 Toolbox running on MATLAB 2019b (the MathWorks) for the EEG analyses. We started with pre-processing the data by applying a fourth-order Butterworth band-pass filter (0.1–100 Hz) on the segmented data of 68 s per segment, hence 2 s before and after sequence onset. Afterwards, we down sampled the data to 256 Hz and re-referenced the channels to a common average of all electrodes. Note that there was no need for eye-blink removal as the participants closed their eyes while listening to the sounds.

#### 2.4.2. Frequency-Domain Analysis

Next, we segmented the pre-processed data again starting after the 2 s fade-in and ending right before the fade-out, at 59.27 s leading to an integer number of oddball (1.333 Hz) cycles (15,172 time bins). We averaged the six trials per condition (Fear, Angry, Happy, Sad, and Scrambled condition) for each participant separately in the time domain to reduce EEG activity not in phase with the auditory stimulation (e.g., noise). We transformed these averages into the frequency domain using a fast Fourier transformation (FFT) and the amplitude spectrum was computed with a high spectral resolution (0.0167 Hz).

The base rate of the voices (4 Hz) and the oddball presentation of emotions (1.333 Hz) and their integer multiples (harmonics) are present in the EEG signal. Responses at these frequencies and their harmonics reflect besides the response to the stimulus presentation and the overall noise. Therefore, we used two measures to describe the response in relation to the noise level: signal-to-noise ratio (SNR) and baseline-corrected amplitudes [43,44]. SNR was computed at each frequency bin as the amplitude value at a given bin divided by the average amplitude of the 20 surrounding frequency bins (i.e., 12 bins on each side, 24 bins, but excluding the two directly adjacent bins and the local minimum and maximum). Baseline-corrected amplitude was computed in a similar way, but here we subtracted the average amplitude of the surrounding bins instead of dividing.

Z-score spectra on group-level data were computed to define the harmonics that were significantly above noise level per stimulation frequency (Z > 1.65 or *p* < 0.05). The z-scores were significant until the 2nd harmonic for the base rate (4 Hz) and until the 4th harmonic for the oddball frequency (1.333 Hz). Those harmonics of the oddball frequency that corresponded to the base frequency (3.999 and 7.998 Hz), were excluded thus the neural responses for oddball stimulation were quantified by summing up the baseline-corrected responses for three harmonics (1.333 Hz, 2.666 Hz, and 5.333 Hz). We used all conditions to determine the number of significant harmonics.

As this is a new paradigm, we wanted to objectively select the regions of interest (ROIs) based on the data of all the subjects. We determined the ROIs separately for the base frequency (4 Hz) and the oddball frequency (1.333 Hz) as we expected different patterns of activation for the different frequencies. We incorporated all conditions including the scrambled one for the ROI delineation. Hence, we calculated the baseline-subtracted amplitude across all subjects for each condition and each electrode, and we summed across the significant harmonics (4 Hz and 8 Hz for the base frequency, and 1.333 Hz, 2.666 Hz, and 5.333 Hz for the oddball frequency). All electrodes for which the baseline-subtracted amplitude of the response was significantly higher than the mean response (Bonferroni corrected) were retained and grouped in an ROI based on their location on the scalp. We extended these ROIs by also including the corresponding contralateral homologue ROIs to investigate potential hemispheric lateralization effects.

#### 2.4.3. Statistical Analyses

Separately for the base rate and oddball responses, a series of linear repeated-measures mixed-models (LMM) were calculated. First, we zoomed in on the contrast between emotion conditions versus scrambled condition across all the electrodes included in the significant core ROIs. Hence, condition (Fear, Angry, Happy, Sad, and Scrambled) and ROI (all core ROIs) were entered as fixed within-subject factor and participant as random factor. Next, we investigated the lateralization of the neural responses comparing only the emotion conditions. Thus, emotion condition (Fear, Angry, Happy, and Sad), ROI (all ROIs including the corresponding contralateral homologue), and emotion × ROI interaction were entered as fixed within-subject factors and participant as random factor. Post-hoc *t*-tests corrected via Holms correction were calculated to assess the significance of particular contrasts.

## 3. Results

### 3.1. Orthogonal Task

We first checked if participants were able to perform the orthogonal task to make sure they were paying attention to the sound streams. The high accuracy of 96.3% (SD = 9.7%) indicated that the participants had no difficulty with the task. Important to note is that there was no significant difference between the conditions in the accuracy of the implicit task, we tested this with a LMM with condition as fixed factor and participant as random factor (*F*(4,76) = 0.31, *p* = 0.86).

### 3.2. Region of Interests

**Figure 3:**
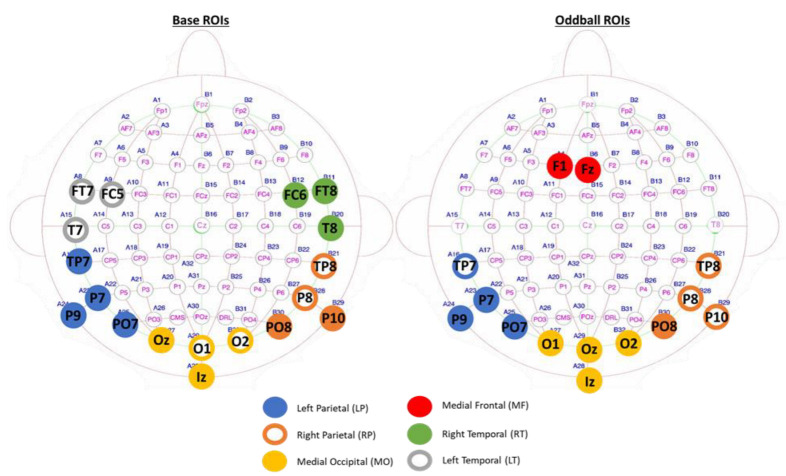
Region of Interest (ROI) delineation for base and oddball responses separately. Significant electrodes and the core ROIs are depicted in full circles and the extended contralateral ROIs are depicted with empty circles.

The explorative investigation of regions of interests resulted in the delineation of 11 significant electrodes for the base frequency (FC6, FT8, Iz, Oz, P10, P7, P9, PO7, T8, TP7, TP8) and 10 significant electrodes for the oddball frequency (F1, Fz, Iz, O1, O2, Oz, P7, P9, PO7, PO8). We divided the significant electrodes in four core ROIs based on the location of the electrodes, three for the base responses: ROI Left Parietal (LP: P7, P9, PO7, TP7), ROI Medial Occipital (MO: O1, O2, Iz, Oz), and ROI Right Temporal (RT: T8, FC6, FT8); and three for the oddball responses: ROI Left Parietal, ROI Medial Occipital, ROI Medial Frontal (MF: Fz, F1). In addition, to investigate possible lateralization effects and to include all significant electrodes, we also included the corresponding contralateral homologue brain areas in our analyses as extended ROIs, thus ROI Right Parietal (RP: P8, P10, PO8, TP8) and ROI Left Temporal (LT: T7, FC5, FT7). Note that O1 and O2 were not significant for the base frequency and TP7 not for the oddball frequency and we still included these electrodes in the ROIs to delimit the number of ROIs needed for the analyses. See Figure 3 for ROI placement for base and oddball separately with contralateral ROIs included.

### 3.3. SNR and Topographies

**Figure 4:**
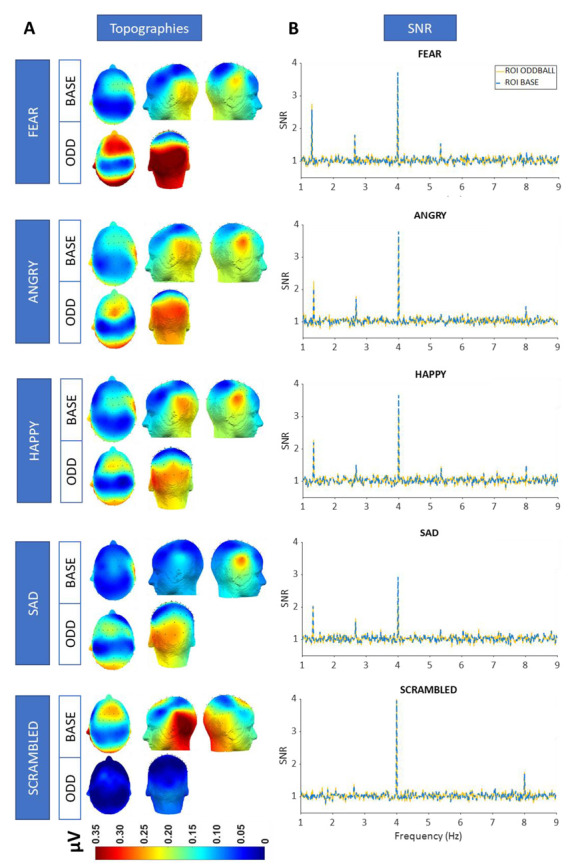
(**A**) Topographies of all conditions showing summed baseline-subtracted averages (in µV) of the significant harmonics, being 4 Hz and 8 Hz for the baseline (BASE) and 1.333 Hz, 2.666 Hz, and 5.333 Hz for the oddball (ODD) frequency. (**B**) Signal-to-noise (SNR) spectra of all conditions, with the blue spectrum representing the responses in the base frequency ROIs (LT, MO, and RT) and the yellow spectrum displaying responses in the oddball frequency ROIs (LP, MO, and MF).

To define the harmonics that were significantly above noise level, we computed Z-score spectra on group-level data for each condition for the base and oddball frequency. We averaged the FFT amplitude spectra across electrodes in the significant regions-of-interest (ROIs) based on the ROI determination and transformed these values into Z-scores. When we look at the signal-to-noise ratio (SNR), we see clear base responses related to the stimulus presentation at the first and second harmonic for every condition (*Z* > 1.65 or *p* < 0.05). At the oddball frequency, we find clear oddball responses at the first, second, and fourth harmonic for each of the emotion conditions (*Z* > 1.65 or *p* < 0.05), but only at the second harmonic for the scrambled control condition. See Figure 4 for the SNR spectra and the topographies of each condition.

Based on visual inspection, for base rate synchronization, we see higher activation at the right side of the brain for the emotion conditions but not for the scrambled control condition. For oddball discrimination, the response was more lateralized to the left side of the brain for the emotion conditions, but no clear response was observable for the scrambled condition.

### 3.4. Contrasting Emotion-Specific Responses Versus Responses for the Scrambled Condition

**Figure 5:**
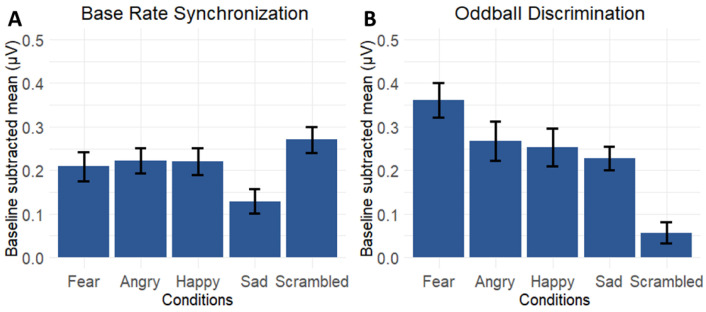
Comparison of the neural responses for the four conditions with emotional utterances and for the scrambled condition. (**A**) Base rate responses with standard error of the mean as the error bar. Summed baseline-corrected amplitudes (µV) at significant base rate harmonics (4 Hz and 8 Hz) and averaged across LP, MO, and RT ROIs reveal that sequences with sad utterances yield lower amplitudes. (**B**) Oddball responses with standard error of the mean as the error bar. Summed baseline-corrected amplitudes (µV) at oddball frequencies (1.333 Hz, 2.666 Hz, and 5.333 Hz) averaged across LP, MO, and MF ROIs reveal that fear discrimination yields the highest amplitudes, and that automatic vocal emotion discrimination is significantly hampered by scrambling the stimuli.

First, we compared the emotion conditions with the scrambled condition to investigate to what extent low-level acoustic features versus high-level emotional characteristics explained by the oddball effect. Figure 5 displays base rate and oddball rate neural responses for the five conditions averaged across the core ROIs yielding significant responses.

An LMM on the base rate responses with condition, ROI, and their interaction as fixed factors and participants as random factor, revealed a significant main effect of condition (*F*(4,266) = 16.97, *p* < 0.0001, partial *η*^2^ = 0.20, 95% CI [0.13, 1]), with post-hoc paired t-testing demonstrating significantly lower responses for the Sad condition as compared to all other conditions (*t*(59) > 5.20, *p* < 0.0001 for all contrasts). We also found a significant main effect of ROI (*F*(2,266) = 6.60, *p* = 0.002, partial *η*^2^ = 0.05, 95% CI [0.01, 1]) and a significant condition by ROI interaction effect (*F*(8,266) = 4.52, *p* = 3.57 × 10^−5^, partial *η*^2^ = 0.12, 95% CI [0.05, 1]). The interaction effect revealed that the amplitudes were equally distributed over the different ROIs for fear, angry, and happy (*t*(19) < 2.23, *p* > 0.110 for all contrasts), but that for the sad condition and the scrambled condition the pattern differed. For the sad condition we found that ROI RT had higher responses in comparison with ROI MO (*t*(19) > 3.12, *p* < 0.017). The scrambled condition showed a different lateralization and we found lower responses at the right, at ROI RT in comparison with the other ROIs (*t*(19) > 5.44, *p* < 0.0001 for both contrasts).

A similar LMM on the oddball discrimination responses revealed an extreme main effect of condition (*F*(4,266) = 41.28, *p* < 0.0001, partial *η*^2^ = 0.38, 95% CI [0.31, 1]), but no effect of ROI (*F*(2,266) = 2.26, *p* = 0.106, partial *η*^2^ = 0.02, 95% CI [0, 1]) nor condition by ROI interaction effect (*F*(8,266) = 0.33, *p* = 0.95, partial *η*^2^ = 9.84 × 10^−3^, 95% CI [0, 1]). Here, post-hoc testing indicated that the amplitude for the scrambled condition was significantly lower than all emotional conditions (*t*(59) > 6.88, *p* < 0.0001 for all contrasts), and the amplitude for the fear condition was significantly higher than all other conditions (*t*(59) > 3.86, *p* < 0.001 for all contrasts).

### 3.5. Investigating Lateralisation Patterns of Emotion-Specific Responses

**Figure 6:**
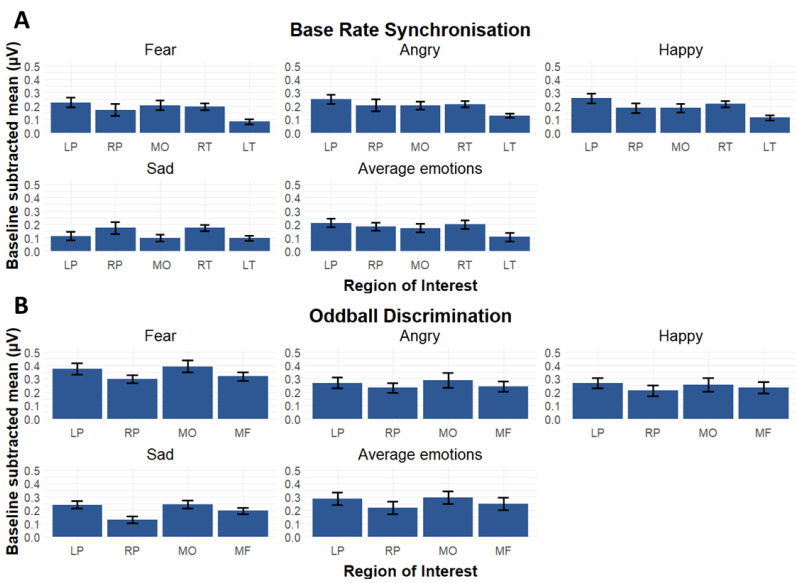
Comparison of the neural responses for the four conditions with emotional utterances as a function of spatial location with standard error of the mean as error bar (ROI). (**A**) Base rate synchronization. Summed baseline-corrected amplitudes (µV) at base rate harmonics reveal that the sad condition yields the lowest responses, and that ROI right temporal (RT) hosts the highest responses. (**B**) Oddball discrimination. Summed baseline-corrected amplitudes (µV) at oddball frequencies reveal that fear and angry yield the highest amplitudes and that ROIs left parietal (LP) and medial occipital (MO) show higher activation than the other ROIs.

To investigate lateralisation patterns of emotion-specific responses, we additionally included the contralateral homologue ROIs in our analyses. Figure 6 displays ROI-specific base rate and oddball rate neural responses for the various emotional conditions. An LMM on the base frequency responses with emotion condition (Fear, Angry, Happy, and Sad), ROI (all base rate ROIs including the corresponding contralateral homologue), and emotion condition × ROI interaction as fixed within-subject factors and participant as random factor revealed a main effect of condition (*F*(3,361) = 8.44, *p* < 0.0001, partial *η*^2^ = 0.07, 95% CI [0.03, 1]) and a main effect of ROI (*F*(4,361) = 12.45, *p* < 0.0001, partial *η*^2^ = 0.12, 95% CI [0.07, 1]), but no significant condition by ROI interaction (*F*(12,361) = 1.55, *p* = 0.10, partial *η*^2^ = 0.05, 95% CI [0, 1]). As expected, the main effect of condition was driven by the lower amplitudes for sad as compared to all other emotions (*t*(99) > 3.52, *p* < 0.003). Post-hoc testing for the main effect of ROI indicated that ROI LT had lower amplitudes than the other significant ROIs (*t*(79) >3.96, *p* < 0.001 for all contrasts) and that ROI LP had higher amplitudes than ROI MO (*t*(79) = 3.05, *p* = 0.019).

A similar condition by ROI LMM on the oddball responses revealed a main effect of condition (*F*(3,285) = 7.20, *p* < 0.0001, partial *η^2^* = 0.17, 95% CI [0.11, 1]),) and a main effect of ROI (*F*(3,285) = 19.62, *p* < 0.0001, partial *η^2^* = 0.07, 95% CI [0.02, 1]), but no ROI by condition interaction effect (*F*(9, 285) = 0.412, *p* = 0.92, partial *η*^2^ = 0.01, 95% CI [0, 1]). The pairwise comparisons revealed that the amplitudes for fear were higher than any other condition (*t*(79) > 4.26, *p* < 0.0001 for all contrasts), and angry had higher amplitudes than sad (*t*(79) = 2.88, *p* = 0.015). Pairwise contrasts for the effect of ROI indicated that ROI LP and ROI MO had both higher amplitudes than ROI RP and ROI MF (*t*(79) > 2.75, *p* > 0.022).

## 4. Discussion

We found clear base and oddball responses for every emotion category, indicating that the brain is able to synchronize with the presentation rate of vocal stimuli and to systematically detect and discriminate subtle vocal emotional utterances from neutral utterances. To ensure that this effect was not purely driven by systematic low-level acoustic differences, we preselected highly heterogeneous vocal stimuli with a high variability in pitch and harmonic ratio, which are important low-level features for emotional voice processing. Yet, despite this huge random variability, pitch and harmonic ratio still varied in a periodic way in the neutral-emotional sound streams, as indicated in Figure 2C. Against this background, it may have been not too surprising to also observe this same periodicity, including the oddball responses, in the EEG spectrum. To further control the relative importance of low-level acoustic features for automatic emotion processing, we also included a scrambled version of the vocal sound streams. This scrambling procedure preserved the low-level spectro-temporal acoustic structure of the sound but ensured that stimuli were no longer recognizable as voices, let alone that the emotional content would have been identified. Preservation of some of the acoustic structure and its periodicity along the vocal sound stream is again demonstrated in Figure 2C. Yet crucially, and importantly, in contrast with the emotion conditions, for the scrambled control condition we only found an EEG base rate response and no selective oddball discrimination responses for the first harmonic (cf. Figure 4). Accordingly, these results clearly indicate that periodicity of low-level acoustic features by itself does not suffice to induce robust oddball EEG responses, but meaningful high-level emotional categories are needed.

For the base rate frequency, we found a main effect of condition with reduced base responses for the sad condition. It appears that sad utterances are confused and misinterpreted often with neutral utterances (16%) and it might be that habituation occurs more pronounced in the sad condition in comparison with the other conditions due the similarity of the neutral and sad utterances leading to lower responses in the EEG data [55]. However, regarding the confusion matrix of the used stimuli (Figure 1), happy utterances were confused with neutral utterances as much as sad utterances (16%) but did not show reduced base responses in comparison with the other conditions. Happy also had the lowest accuracy of all emotions, which is also supported by other vocal emotion studies [15].

While comparing the oddball response between the different emotions, we observed the highest response for the detection and discrimination of fearful and angry voices. This echoes the general observation that threat-related emotions, such as fear and anger, may be important from an evolutionary perspective to survive unknown situations, and may, therefore, be most easily detected and attract our attention [22]. This finding neatly aligns with a similar observation showing the highest frequency-tagged EEG discrimination responses for visually presented emotional expressions of fearful and angry faces in a continuous stream of neutral faces [47]. In this study, despite the difference in modality, a similar pattern of emotion discrimination responses was observed, with fearful and angry expressions eliciting the strongest response, happy expressions an intermediate response, and sad facial expressions the lowest response.

We did not observe a condition (fear, angry, happy, and sad) by ROI interaction effect, nor for the base rate responses nor for the oddball responses, suggesting the presence of a similar neural activation pattern for all emotions used in the experiment. However, note that EEG, as compared to MRI, is not the most sensitive method to detect small spatial differences in activation patterns. For the base frequency, which indices the periodic presentation of voices, we found a main effect of ROI, revealing a right-side lateralisation of activity at the temporal cortex. Importantly, the scrambled condition, which involved the presentation of non-recognizable artificial sounds, did not display this right lateralisation at base frequency. This echoes the general literature that voice processing, and certainly emotional voice processing, is right lateralised [23,24]. On the other hand, the absence of a right lateralization for the 4 Hz base rate of the scrambled condition does not corroborate the asymmetric temporal sampling in time theory of Poeppel and colleagues [27], which postulates that slower oscillations (~200 ms) are preferentially processed by the right auditory cortex.

In response to the oddball frequency, we found a different lateralisation pattern, revealing higher amplitudes at the left side of the brain, in particular in the left parietal cortex. The left lateralisation of these emotional voice discrimination responses may be related to the higher difficulty level of the task, as differentiating emotions is harder than simple voice processing, and studies have demonstrated more left lateralized brain activation for tasks that are more difficult [26,56]. For both the base and the oddball responses, we also found activation in posterior occipital regions and even some medial frontal activation for the oddball response. This pattern may originate from activity in the auditory cortex and posterior STS projecting towards the posterior and anterior regions of the scalp because of the particular folding of the gyri. However, to pinpoint the exact spatial location and source of these responses, methods with a higher spatial resolution would be required.

We want to point out that every study has its limitations. First, EEG is not appropriate to study the fine-grained spatial location of brain responses involved in emotion discrimination, hence an MRI alternative might be more suited for an in-dept investigation of possible lateralization effects. Next, as we only included male adults in the present study, it is not possible to generalize the findings to the general population. In this regard, it would be insightful to extend this study to a group of female participants. It would be particularly interesting to directly compare neural responses of female and male participants to investigate if the classical gender differences in vocal emotion recognition [51] are still present in this implicit paradigm where explicit recognition and naming of the emotion is not required. Furthermore, as this paradigm taps into automatic and implicit emotional processing, it would be very well suited for applications in child and even infant populations, to investigate developmental trajectories of vocal emotion processing. Also, exploration of emotion processing sensitivity in populations with known socio-communicative difficulties such as autism [57] would be highly relevant.

Another possible concern of the present study is that we did not use naturally produced vocal emotional utterances, but smaller speech segments extracted from vocal utterances that were produced by actors who imitated a certain emotion. It can be questioned whether intentionally produced (or acted) vocal emotional utterances display similar characteristics as naturally produced emotional utterances. This is an issue in emotion research in general. However, whereas acted facial expressions can indeed be differentiated from genuine facial expressions [58], research has shown that listeners are not able to differentiate whether a vocal emotional utterance was acted or naturally produced [59]. This is important as it validates and justifies the use of acted utterances. Accordingly, due to the lack of validated databases with naturalistic emotional utterances, thus far, we have to work with ‘acted’ databases. In this regard, the newly developed EVID database can play an important role in vocal emotional research, as it strives to be as realistic as possible and uses short clips of sentences which are still recognizable in terms of emotional valence.

## 5. Conclusions

Overall, we demonstrated that we can track the discrimination and categorization of complex vocal emotional utterances with frequency tagging EEG and that these emotion-selective responses are at least partially independent from low-level acoustic features (see also [49]). This fast, straightforward, and double-objective approach offers a unique and powerful tool to quantify the implicit sensitivity for subtle vocal emotional cues at the individual subject-level, without any overt behavioral processing. This opens up the way to apply this paradigm to investigate emotion processing abilities in young children and infants that are unable to understand instructions or provide explicit responses, and to investigate particular clinical populations that are characterized by atypical emotion processing abilities, such as autism spectrum disorder, schizophrenia, frontotemporal dementia, anxiety disorder, etc. Also, at a more fundamental level, it paves the way for implementing other complex sound categorization frequency-tagging paradigms (pinpointing for instance vocal identity discrimination), thereby contributing to an advanced understanding of human auditory categorization in general.

## Data Availability

The newly designed Emotional Voices and Identity Database (EVID), created for this study and described in the manuscript, will be made publicly available, as well as the analysis scripts. The EEG data of the study will be available anonymized upon request.

## Supplementary Materials

The following supporting information can be downloaded at: https://www.mdpi.com/article/10.3390/brainsci13020162/s1, Figure S1: Low-level features of the vocal utterances. Low-level features are plotted for every single scrambled stimulus of every emotion condition. On the left the pitch (f0, fundamental frequency) is plotted and on the right harmonic ratio (hr in %). Note the large overlap with the original stimuli.
