# Supplementary material for: The Sound of Emotion: Pinpointing Emotional Voice Processing Via Frequency Tagging EEG
Source: Brain Sci. 2023 Jan 18;13(2):162. doi: 10.3390/brainsci13020162 (PMC9954097; doi:10.3390/brainsci13020162)
Supplement: Supplementary file 1 [file brainsci-13-00162-s001.zip › brainsci-2120938-supplementary.pdf]

Supplementary

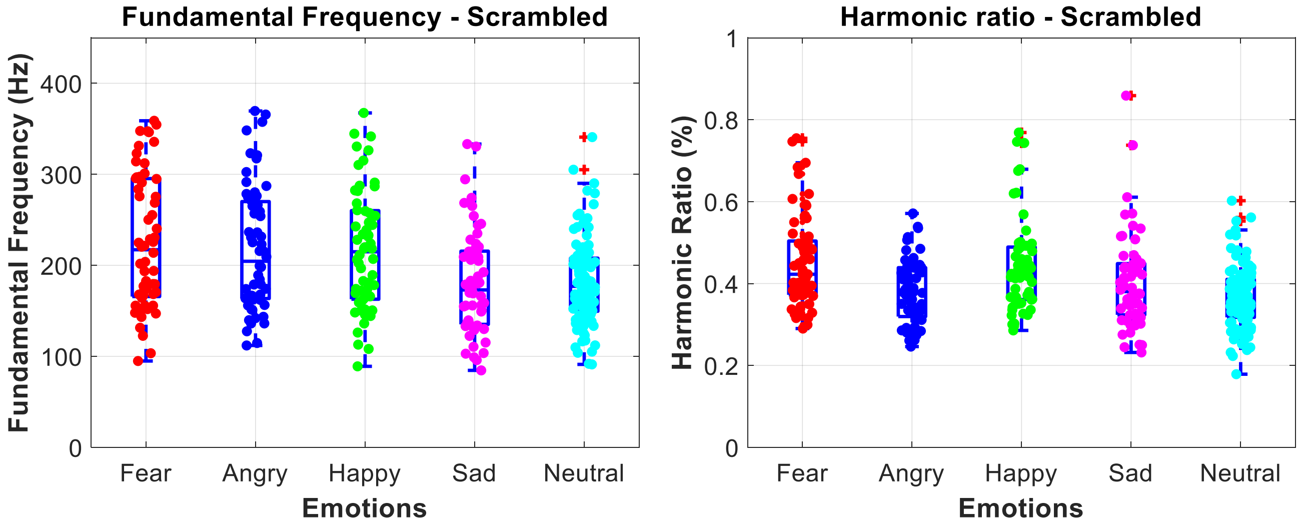

Figure S1. ***Low-level features of the vocal utterances***. *Low-level features are plotted for every single scrambled stimulus of every emotion condition. On the left the pitch (f0, fundamental frequency) is plotted and on the right harmonic ratio (hr in %). Note the large overlap with the original stimuli.*
